# The paracrine effects of adipocytes on lipid metabolism in doxorubicin-treated triple negative breast cancer cells

**DOI:** 10.1080/21623945.2021.1979758

**Published:** 2021-11-23

**Authors:** Ilze Mentoor, Anna-Mart Engelbrecht, Mari van de Vyver, Paul J. van Jaarsveld, Theo Nell

**Affiliations:** a Department of Physiological Sciences, Faculty of Science, University of Stellenbosch, Stellenbosch, South Africa; b African Cancer Institute (ACI), Department of Global Health, Faculty of Medicine and Health Sciences, Stellenbosch University, Stellenbosch, South Africa; c Division of Clinical Pharmacology, Department of Medicine, Faculty of Medicine and Health Sciences, Stellenbosch University, Stellenbosch, South Africa; d Non-Communicable Diseases Research Unit, South African Medical Research Council, Cape Town, South Africa; e Division of Medical Physiology, Faculty of Medicine and Health Sciences, Stellenbosch University, Stellenbosch, South Africa; f Centre for Cardio-Metabolic Research in Africa (CARMA), Department of Biomedical Sciences, University of Stellenbosch, Stellenbosch, South Africa

**Keywords:** Adipocytes, breast cancer, lipolysis, fatty acids, inflammation, treatment resistance

## Abstract

Adipocytes in the breast tumour microenvironment promotes acquired treatment resistance. We used an in vitro adipocyte-conditioned media approach to investigate the direct paracrine effects of adipocyte secretory factors on MDA-MB-231 breast cancer cells treated with doxorubicin to clarify the underlying treatment resistance mechanisms. Cell-viability assays, and Western blots were performed to determine alterations in apoptotic, proliferation and lipid metabolism protein markers. Free fatty acids (FFA) and inflammatory markers in the collected treatment-conditioned media were also quantified. Adipocyte secretory factors increased the cell-viability of doxorubicin-treated cells (p < 0.0001), which did not correspond to apoptosis or proliferation pathways. Adipocyte secretory factors increased the protein expression of hormone-sensitive lipase (p < 0.05) in doxorubicin-treated cells. Adipocyte secretory factors increased the utilization of leptin (p < 0.05) and MCP-1 (p < 0.01) proteins and possibly inhibited release of linoleic acid by doxorubicin-treated cells (treatment-conditioned media FFA profiles). Adipocyte secretory factors induced doxorubicin treatment resistance, by increasing the utilization of inflammatory mediators and inhibiting the release of FFA by doxorubicin-treated cells. This further promotes inflammation and lipid metabolic reprogramming (lipid storage) in the tumour microenvironment, which breast cancer cells use to evade the toxic effects induced by doxorubicin and confers to acquired treatment resistance.

## INTRODUCTION

Globally, breast cancer is the most prevalent type of cancer diagnosed in women [1,2], of which triple-negative breast cancer (TNBC) accounts for 15–20% of all cases [3]. TNBC is known for its aggressive nature and metastasis, as well as poor clinical and treatment outcomes compared to other breast cancer subtypes [4]. Effective treatment strategies are limited for TNBC with approximately only 20% of cases responding to anti-cancer treatment [5], highlighting the importance of identifying underlying molecular mechanisms responsible for decreased treatment efficacy in TNBC. Doxorubicin is an anti-neoplastic agent commonly used in the treatment of TNBC [6]. However, doxorubicin’s use as a chemotherapeutic agent is associated with many cellular toxicities and the development of treatment resistance [7]. Furthermore, pre-clinical evidence identifies obesity as a key role player in breast cancer chemotherapeutic drug resistance [8–10]. This is of clinical significance for overweight/obese TNBC breast cancer patients receiving chemotherapeutic agents such as doxorubicin, who are already disadvantaged by a limited treatment efficacy [5].

TNBC develops independent of hormone-related pathways and existing evidence proposes that paracrine interaction between tumour cells, adipocytes, macrophages, fibroblast as well as endothelial cells within the tumour microenvironment favours cancer cell survival [11–14]. Adipocytes secrete various soluble factors including, adipokines, cytokines, chemokines and matrix metalloproteases, which have been linked to tumour progression [3,15–17]. For example, adipocyte-derived interleukin-6 (IL-6), interleukin-1β (IL-1β), leptin, resistin and macrophage chemoattractant protein-1 (MCP-1) increased the proliferation, migration and metastasis of MDA-MB-231 cells [18–21]. Adipocytes in the TME also promote extracellular matrix degradation and remodelling *via* increased secretion of chemokine ligand-5 and matrix metallopeptidase-9, which promotes metastasis in MDA-MB-231 cells [22,23]. These adipocyte-derived adipokines are also proposed to bind to their respective receptors on cancer cells, activating various downstream signalling pathways (i.e. Phosphoinositide-3-kinase (PI3K)/Akt and MAP kinase (MAPK) pathway) which regulate cancer cell proliferation, migration and apoptosis [24]. Additionally, newer evidence points to adipocyte-derived fatty acids (FA) favouring breast cancer cell survival [15,25,26].

To date, several studies investigated the mechanisms underlying the role of adipocytes/adipose tissue in breast cancer treatment resistance. Firstly, adipocytes were shown to metabolize Daunorubicin and decrease its efficacy in leukaemia cells [27]. Doxorubicin resistance has also been the result of adipocyte-derived resistin modulating cell-death pathways [28]. More recently, 29 showed that adipocytes promoted doxorubicin treatment resistance in various breast cancer cell lines by promoting the cellular efflux of doxorubicin from the nuclease to cytoplasmic vesicles. This process was mediated by a major vault protein, responsible for the intracellular transport of molecules including chemotherapeutic drugs. Interestingly, the exportation of doxorubicin-vesicles *via* the major vault protein-mediated mechanism was exacerbated by obesity [29]. Previous work in our group showed that diet-induced obesity attenuated doxorubicin’s efficacy in TNBC tumours by inducing differential expression of lipid metabolic markers in mammary and tumour tissue, which altered both the tumour phospholipid and mammary adipose tissue FA profiles [10]. This results in oncogenic driven metabolic reprogramming, another resistance mechanism cancer cells employ to sustain their survival, high-energy demand and proliferative nature [30].

Furthermore, breast cancer cells disrupt normal lipid metabolism by increasing *de novo* synthesis of FA [31–33]. Additionally, through paracrine effects, breast cancer cells also increase the uptake of exogenous FA by inducing lipolysis in adipocytes by increasing the expression of lipolytic proteins (i.e. adipose triglyceride lipase (ATGL) and hormone-sensitive lipase (HSL)), all creating a continuous release of free fatty acids (FFA) [25,34]. In agreement, adipocytes in the TME are characterized by morphological and phenotypical changes evident by a fibroblast-like morphology [18,35] and decreased lipid droplet size and content, which is more profound in MDA-MB-231 compared to MCF-7 breast cancer cells [35,36].

Balaban and colleagues, showed that these adipocyte-derived FFA are taken up by breast cancer cells *via* increased expression of carnitine palmitoyl transferase I, which increased cellular proliferation and migration [25]. Additionally, fatty acid-binding protein-4 and −5 (which act as intracellular lipid chaperones), fatty acid translocase (transmembrane channel responsible for exogenous FA uptake) [37–40], and carnitine palmitoyl transferase I (involved in β-oxidation) have all been shown to be increased in TNBC cell lines [36,41,42]. It was also demonstrated that FFA availability was exacerbated in ‘obese’ adipocytes, and that excessive FFA uptake by breast cancer cells favoured proliferation and migration [36], which implicates obesity as a driving factor of metabolic reprogramming [43,44].

Therefore, to elucidate whether adipocytes play a role in acquired treatment resistance in TNBC cells, we used an *in vitro* model to investigate the direct effects of adipocyte secretory factors on MDA-MB-231 cells treated with doxorubicin using a conditioned media approach and to elucidate potential underlying mechanisms.

## METHODS

### Culturing of Human Adipose Tissue Derived Stem Cells

The human adipose tissue-derived stem cell (hADSC) line (hADSCs; Donor 26,508, #0000364977, Poietics, Lonza, Basel, Switzerland) was used for adipogenic differentiation into mature adipocytes (Supplementary Figure 1). The hADSCs were cultured in 100 mm tissue culture dishes (55 cm^2^, SPL, Life Sciences, Korea) under standard incubator conditions (37°C and 5% CO_2_ humidity) in growth media.

**Figure 1.**
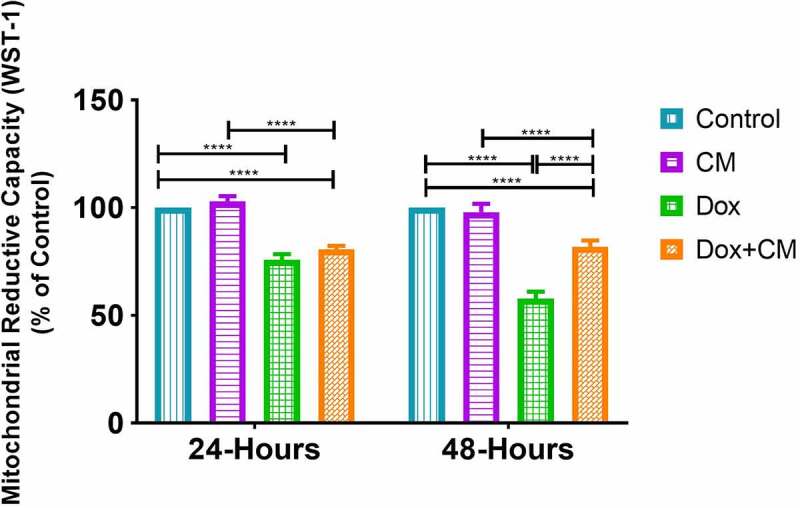
The effect of adipocyte secretory factors in conditioned-medium on cell-viability in MDA-MB-231 triple negative breast cancer cells treated with/without doxorubicin for after 24 hours and 48 hours. Results are presented as mean ± SEM (n = 3). Two-way ANOVA with Fishers LSD post hoc correction was employed. p < 0.05 was considered as statistically significant. **** = p < 0.0001. Control, normal growth media; CM, 30% adipocyte-conditioned media and 70% growth media ratio; Dox, 2.5 µM Doxorubicin; Dox+CM, 2.5 µM Doxorubicin + CM (30% adipocyte-conditioned media and 70% growth media ratio)

The growth media consisted of Dulbecco’s Modified Eagle’s medium (DMEM) with high-glucose (4.5 g/L) and ultra-glutamine (Life Technologies, California, USA), supplemented with 10% Foetal Bovine Serum (FBS) (Scientific Group, South Africa), and 1% Penicillin Streptomycin (PenStrep) (Life Technologies, California, USA). Growth media were replaced regularly until cells were sub-cultured upon reaching 70–80% confluency. The cell monolayer was washed with pre-heated phosphate buffered saline (PBS), followed by an incubation step with 0.5% Trypsin EDTA (Life Technologies, California, USA) until detachment of cells from the surface of the dish. The cell suspension was centrifuged (5 min @ 250 RCF (g)), and the pellet re-suspended in fresh growth media for seeding at a density of 5 000 cells/cm^2^. Passage number for hADSC differentiation ranged from 4 to 6.

### Adipogenesis and Collection of dipocyte-Conditioned Medium

The sub-cultured hADSCs were allowed to reach 2 days’ post confluence (passage 4–6) before differentiation was initiated (Supplementary Figure 1). Adipogenic differentiation medium (AM) consisted of DMEM (Life Technologies, California, USA), supplemented with 10 µM insulin (Sigma-Aldrich®, Missouri, USA), 0.5 mM 3-isobutyl-1-methylxanthine (IBMX) (Sigma-Aldrich®, Missouri, USA), 1 µM dexamethasone (Sigma-Aldrich®, Missouri, USA), 56 µM indomethacin (Sigma-Aldrich®, Missouri, USA), and 1 ml FBS (Scientific Group, South Africa) [45]. AM was replaced every second day for a period of 14 days.

To collect adipocyte-conditioned media, AM was discarded and replaced with fresh growth media (DMEM with high glucose (4.5 g/L), 10% FBS and 1% PenStrep) for 24 hours (Supplementary Figure 1). After 24 hours, the media was collected (referred to as adipocyte-conditioned media), pooled and filtered (0.2 µm filter). The adipocyte-conditioned media was snap frozen in liquid nitrogen and stored at −80°C. The differentiation protocol was repeated six times to create enough adipocyte-conditioned media for subsequent experiments. Following the collection of adipocyte-conditioned media (prior to its use), successful differentiation of mature adipocytes was confirmed by quantitation of triglycerides by the Oil Red O staining method as previously described [45]. Mature adipocytes were stained with Oil Red O stain (0.7% Oil Red diluted in 70% (v/v) isopropanol), 30-minute incubation at room temperature (22°C). Cells were washed three times with distilled water to remove any residual stain and at least four random images were taken per dish using a light microscope (Olympus CKX41, CachN 10/0.25 PhP objective) and EOS600D Canon digital camera.

### Breast Cancer Cell Culturing

#### Experimental Protocol

A human adenocarcinoma TNBC cell line (MDA-MB-231) was used and cultured in growth medium (DMEM, 10% FBS and 1% PenStrep). Once cultures reached 70–80% confluency, cells were sub-cultured and/or seeded as previously described. For each experiment, MDA-MB-231 cells were seeded (250,000 cells per well) into 6-well tissue culture plates (9.5 cm^2^, SPL Life Sciences, Korea), and allowed to grow and attach. Prior to any treatment, growth medium (DMEM, 10% FBS, 1% PenStrep) was aspirated and the cells washed with pre-heated PBS to remove all cellular debris. The experimental treatment protocol included four experimental treatment groups: (i) control, (ii) adipocyte-conditioned media (CM), (iii) doxorubicin (Dox), and (iv) a combination of adipocyte-conditioned media and doxorubicin (Dox+CM) (Table 1). The adipocyte-conditioned media treatment groups consisted of 30% adipocyte-conditioned media and 70% growth media ratio [46,47], to maintain cellular-viability and to still elicit the desired response.

**Table 1.** Experimental treatment protocol

EXPERIMENTAL TREATMENT PROTOCOL		
Treatment Groups	Treatment	Duration
Control	Growth media: DMEM, 10% FBS, 1% PenStrep	48 Hours
Adipocyte-Conditioned Media (CM)	30% Adipocyte-conditioned media and 70% growth media ratio	48 Hours
Doxorubicin (Dox)	Growth media: DMEM, 10% FBS, 1% PenStrep and Doxorubicin (2.5 µM)	48 Hours
Adipocyte-Conditioned Media + Doxorubicin (Dox+CM)	30% Adipocyte-conditioned media and 70% growth media ratio and Doxorubicin (2.5 µM)	48 Hours

Following the respective treatments (48 hours), all plates were placed on ice and cell culture media (supernatant) collected and filtered (0.2 µm filter); now referred to as treatment-conditioned media. The treatment-conditioned media was snap frozen in liquid nitrogen and stored at −80°C until subsequent adipokine and free fatty acid analysis. All experimental protocols were performed in triplicate with (n = 3) replicates per biological repeat.

### Doxorubicin Treatment

The optimal concentration of 2.5 µM doxorubicin treatment dosage was selected based on assessment by dose–response experiments and cell-viability assays previously established [48]. The stock solution of doxorubicin hydrochloride (D5794, LKT® laboratories, Minnesota, USA) was dissolved in DMEM, and the desired concentration of doxorubicin was diluted into complete growth/treatment media before each experiment.

### In Vitro *Model Analyses*

#### Cell-Viability Assay

The WST-1 assay (Roche®, Merck®, #5,015,944,001) was used to assess metabolic active cells according to manufacturer’s specifications. Briefly, MDA-MB-231 TNBC cells were seeded (25,000 cells per well) into 48-well plates (0.95 cm^2^, SPL Life Sciences, Korea), and incubated with 250 μl growth medium (DMEM, 10% FBS, 1% PenStrep), followed by subsequent treatment (Table 1). Following treatments, the media in each well was replaced with fresh growth media containing a 10% WST-1 solution. Cells were incubated for 85 minutes at a 37°C and 5% CO_2_ humidity (C01901R, Snijders Scientific, the Netherlands), followed by a one-minute shaking step at 22°C, until all formazan crystals dissolved. Experiments were performed under dark conditions with plates covered with aluminium foil. Absorbance was measured at 440 nm (EL800, Bio-Tek Instruments Inc. Vermont, USA). All experimental treatment groups were analysed in triplicate in three independent experiments. Absorbance values were expressed as a percentage of the control group *versus* the treated control group. The optimal treatment duration for all experimental groups following treatment was based on assessment by cell-viability assays.

### Protein Analysis and Western Blots

All tissue culture plates were placed on ice, and the culture media was collected (referred to as treatment conditioned media). Plates were washed with PBS three times to remove cellular debris. Cold modified Radio-immunoprecipitation assay buffer (RIPA) (80 µl), containing protease and phosphatase inhibitors (2.5 mM Tris-HCL, 1 mM EDTA, 1 mM dithiothreitol, 0.1 mM phenylmethylsulfonyl fluoride, 1 mM benzamidine, 50 mM NaF, 4 mg/ml SBTI, 10 mg/ml leupeptin, 0.1% SDS, 0.5% sodium deoxycholate and 1% NP40, calibrated to pH 7.4), was added to each plate, followed by a 5-minute incubation on ice. Cells were detached from the flask/plate using a sterile cell scraper rinsed with 100% ethanol between all samples. Whole cell lysates were collected from the plate, sonicated three times at 3 Hz for 3 s each, and centrifuged at 35,000 RCF (g) for 5 minutes at 4°C. The supernatant was collected into Eppendorf tubes and were run through Amicon® Ultra 0.5 ml filters (Merck, Darmstadt, Germany) for protein purification and stored at −80°C. A Direct Detect® infrared spectrometer (DDHW00010-WW, Merck, Darmstadt, Germany) was used to quantify the protein content of samples.

Sample preparation of protein aliquots containing 30 µg protein was diluted with Laemmli sample buffer and boiled for 5 minutes to denature proteins before being loaded into 4–15% polyacrylamide fast cast gels (mini-PROTEAN® TGX™ Gels, Bio-Rad) for separation by SDS-PAGE. Gels were run at 110 V (constant) and 400 mA for approximately 120 minutes (Power Pac 300, BioRad). The electro-transfer of proteins from the gel to the prepared PVDF membranes was achieved using a semi-dry electro-transfer system (TransBlot® Turbo™ v1.02, BioRad) for 30 minutes at 25 V and 1.0 A. Transfer efficiency was evaluated using the stain-free blot protocol provided on a Chemi-Doc™ MP (BioRad) system. All the membranes were washed with 0.1% Tris Buffered Saline-Tween 20 (TBS-T) and blocked for 1 hour in 5% (w/v) non-fat milk and TBS-T at room temperature, to prevent non-specific binding. The PVDF membranes were then incubated at 4°C overnight with a primary antibody (see Supplementary Table 1).

The membranes were washed three times for 5 minutes each with TBS-T, prior to incubation with anti-rabbit/mouse IgG horseradish peroxidase conjugated secondary antibody (1:10,000) (Cell Signalling Technologies), for 1 hour at 22°C. This was followed by a wash step in TBS-T (five times for 5 minutes each), before specific bands were visualized (ImageLab 4.0 software on a Chemi-Doc™ MP, BioRad, USA), and detected using the Enhanced chemiluminescence western blotting substrate detection kit (BioRad, USA). Protein quantification of samples was normalized to total protein signal in each lane present on the same membrane after blotting, as determined by the Stain-Free™ (ImageLab 4.0 software, Biorad USA), properties of the blot and is expressed as a percentage of the control. Cleavage and phosphorylation protein expressions are represented as a fold change.

### Quantification of Extracellular Inflammatory Markers Released by MDA-MB-231 cells in the Collected Treatment-Conditioned Media

The collected treatment-conditioned media from all experimental treatment groups were used to quantify the extracellular concentration levels of adiponectin, leptin, IL-1β, resistin and MCP-1 using a custom panel milliplex luminex kit (HADCYMAG-61 K, Merck, Darmstadt, Germany). Analytes were measured simultaneously using the MAGPIX-system plate reader (Bio-Rad, APX1042) and concentrations (expressed in pg/ml) were reported on Bioplex Software 6.1.

### Analysis of Collected Treatment-Conditioned Media to Determine the Extracellular Free Fatty Acid Profile of MDA-MB-231 cells

Lipids, from 1 ml aliquots of treatment-conditioned media were extracted with chloroform: methanol (21 ml; C:M; 2:1; v:v; Sigma-Aldrich®, Missouri, USA) containing 0.01% butylated hydroxytoluene (antioxidant) by applying an adapted extraction method [49]. Briefly, extraction solvents were added to each sample followed by a 20-minute shaking step. Saline saturated with chloroform: methanol: saline (4.2 ml; CMS; 86:14:1; v:v:v; Sigma-Aldrich®, Missouri, USA) was added, mixed and centrifuged at 60 RCF (g) for 10 minutes at 4°C. The bottom phase was collected and transferred to a 12 ml glass tube with a screw cap. To the remaining upper layer, 10 ml CMS was added and was vigorously shaken for 1 minute using a vortex mixer followed by a centrifugation step. The bottom layer was collected and combined with the first, partially evaporated collection, and further evaporated to dryness under nitrogen gas-flow and water bath set at 37°C.

The FFA fraction was separated from other lipid fractions using thin-layer chromatography (TLC) silica gel 60 plates (10 × 10 cm; No. 1.05626.0001; Merck, Darmstadt, Germany), and eluted with the solvent system petroleum ether (B&M Scientific, Cape Town, South Africa): diethyl ether (Merck, Darmstadt, Germany): acetic acid (Merck, Darmstadt, Germany) (90:30:1; v:v:v). The lipid band containing the separated FFA fraction was demarcated by visualization under long-wave UV light after plates were sprayed with chloroform: methanol (1:1; v:v) containing 2,5-bis-(5ˊ-tert-butylbenzoxazolyl-[2ˊ]) thiophene (10 mg/100 ml; Sigma-Aldrich®, Missouri, USA). These regions were scraped off the plates into glass tubes with screw caps followed by FA methyl esters (FAMEs) production by adding 2 ml methanol: sulphuric acid (H_2_SO_4_; BDH Chemicals, Poole, England) (95:5; v:v) and applying heat (70°C) for 2 hours. After cooling, the resulting FAMEs were extracted with 1 ml water and 3 ml *n*-hexane (Sigma-Aldrich®, Missouri, USA). The upper hexane layer was collected and evaporated to dryness. The FAMEs were re-dissolved in a small volume of *n*-hexane and analysed (sample injection volume 1 µl) by Gas-liquid chromatography (GLC) (Trace 1300 Gas Chromatograph; Thermo Fisher Scientific, MA, USA) equipped with a flame-ionization detector and a 30 m capillary column of 0.32 mm internal diameter (BPX70 0.25 µm; SGE International Pty Ltd, Victoria, Australia). Gas flow rates were as follows: N_2_ (make up gas), 25 ml/min; synthetic air, 250 ml/min; H_2_, 25 ml/min, with a split ratio of 15:1 and column flow (H_2_, carrier gas) was set at 1.0 ml/min. Oven temperature programming was linear at 3.5°C/min, initial temperature 140°C (hold-time 1 min), final temperature 220°C (hold-time 5 minutes), injector temperature 220°C, and detector temperature 250°C.

**Figure 2.**
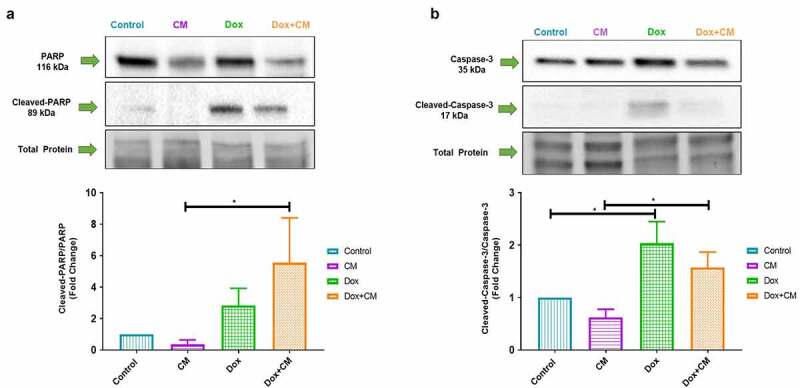
The effect of adipocyte secretory factors in conditioned-medium on (a) PARP and (b) caspase-3 protein cleavage in MDA-MB-231 triple negative breast cancer cells treated with/without doxorubicin for 48-hours. Results are presented as mean ± SEM, and as a fold change (n = 3 PARP and n = 4 Caspase-3). One-way ANOVA with Fishers LSD post hoc correction was employed. p < 0.05 was considered as statistically significant. * = p < 0.05. Control, normal growth media; CM, 30% adipocyte-conditioned media and 70% growth media ratio; Dox, 2.5 µM Doxorubicin; Dox+CM, 2.5 µM Doxorubicin + CM (30% adipocyte-conditioned media and 70% growth media ratio)

The FAMEs were identified by comparing the retention times with those of the standard FAME mixture (27 FAMEs, NuChek Prep Inc., Elysian, MN, USA). The individual FAMEs were quantified using heptadecanoic acid (C17:0; Sigma-Aldrich®, Missouri, USA) as an internal standard and the results are expressed as total µg FAMEs/ml treatment conditioned medium (Supplementary Figure 3a). In addition, individual FAME mass was expressed as a percentage of the total mass of FAMEs (Figure 2 and 6 A-E; Supplementary Figure 4a).

**Figure 3.**
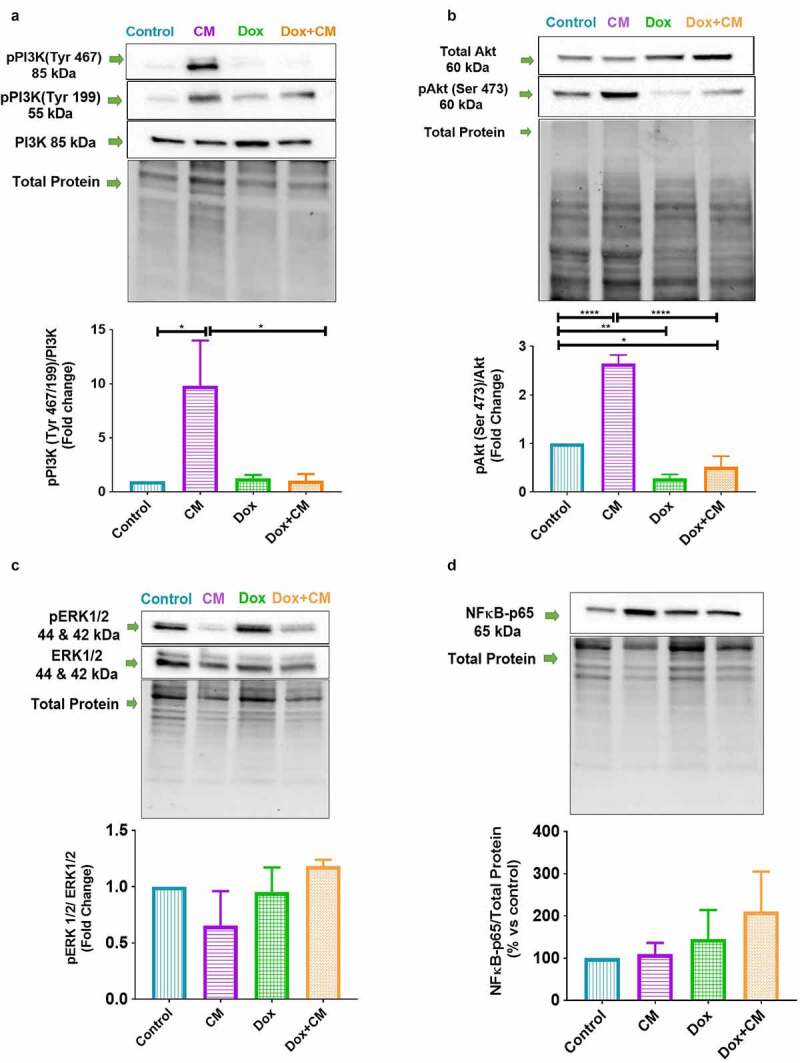
The effect of adipocyte secretory factors in conditioned-medium on proliferation pathways. (a) PI3K phosphorylation (Tyr 467, Tyr 199), (b) Akt phosphorylation (Ser 473), (c) ERK1/2 phosphorylation (pERK1, Thr 202/Tyr 204; pERK2, Thr185/Tyr187) and (d) NFĸB-p65 protein expression in MDA-MB-231 triple negative breast cancer cells treated with/without doxorubicin for 48-hours (n = 3). Results are presented as mean ± SEM and as a fold change (except for NFĸB-p65). One-way ANOVA with Fishers LSD *post hoc* correction was employed. p < 0.05 was considered as statistically significant. * = p < 0.05, ** = p < 0.01, and **** = p < 0.0001. Control, normal growth media; CM, 30% adipocyte-conditioned media and 70% growth media ratio; Dox, 2.5 µM Doxorubicin; Dox+CM, 2.5 µM Doxorubicin + CM (30% adipocyte-conditioned media and 70% growth media ratio)

**Figure 4.**
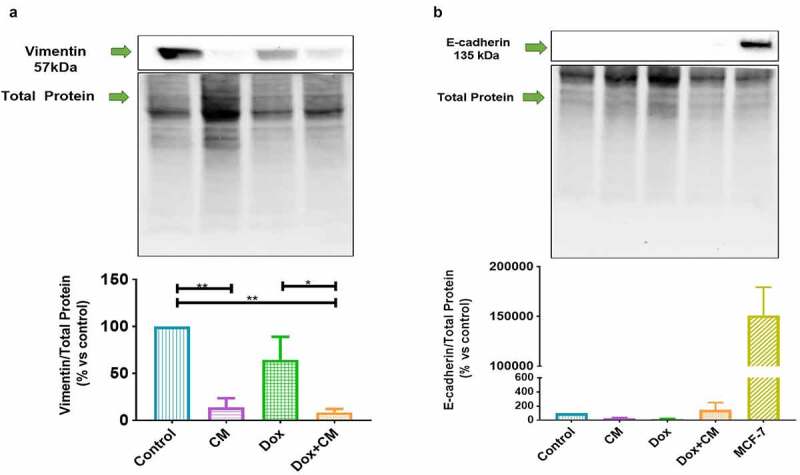
The effect of adipocyte secretory factors in conditioned-medium on EMT markers in MDA-MB-231 triple negative breast cancer cells treated with/without doxorubicin for 48-hours. (a) Vimentin, and (b) E-cadherin. Results are presented as mean ± SEM (n = 3). One-way ANOVA with Fishers LSD *post hoc* correction was employed. p < 0.05 was considered as statistically significant. * = p < 0.05 and ** = p < 0.01. Control, normal growth media; CM, 30% adipocyte-conditioned media and 70% growth media ratio; Dox, 2.5 µM Doxorubicin; Dox+CM, 2.5 µM Doxorubicin + CM (30% adipocyte-conditioned media and 70% growth media ratio)

**Figure 5.**
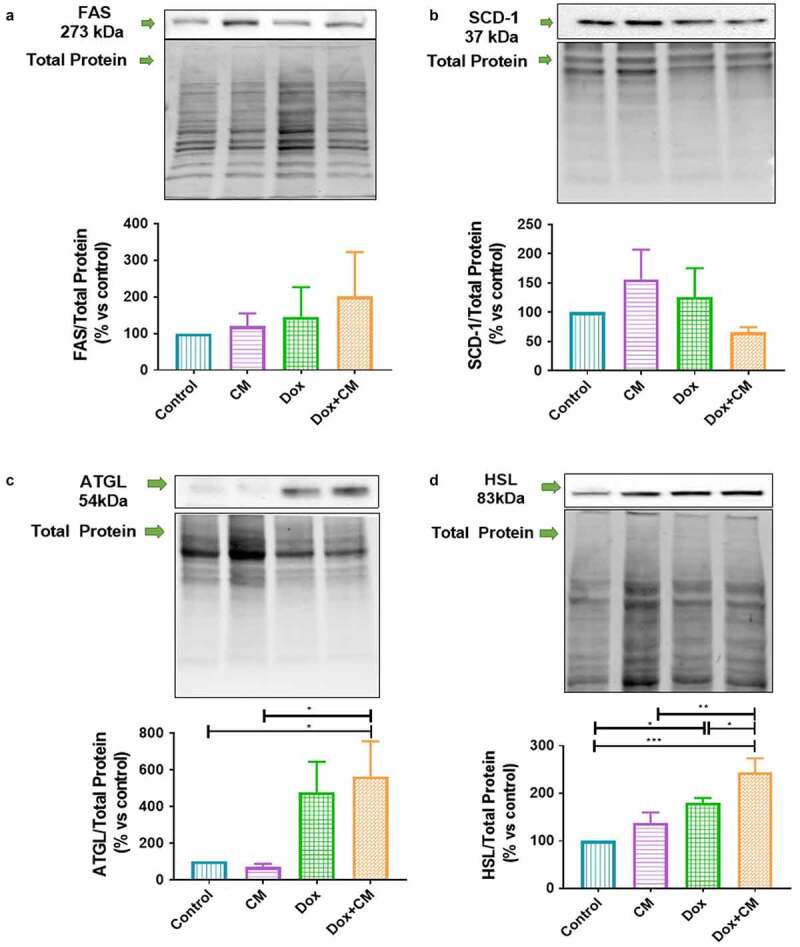
The effect of adipocyte secretory factors in conditioned-medium on markers of lipid metabolism in MDA-MB-231 triple negative breast cancer cells treated with/without doxorubicin for 48-hours (a) FAS, (b) SCD-1, (c) ATGL, and (d) HSL. Results are presented as mean ± SEM (n = 3, except HSL n = 4). One-way ANOVA with Fishers LSD *post hoc* correction was employed. p < 0.05 was considered as statistically significant. * = p < 0.05, ** = p < 0.01 and *** = p < 0.001. ATGL, Adipose triglyceride lipase; FAS, Fatty acid synthase; Control, normal growth media; CM, 30% adipocyte-conditioned media and 70% growth media ratio; Dox, 2.5 µM Doxorubicin; Dox+CM, 2.5 µM Doxorubicin + CM (30% adipocyte-conditioned media and 70% growth media ratio)

**Figure 6.**
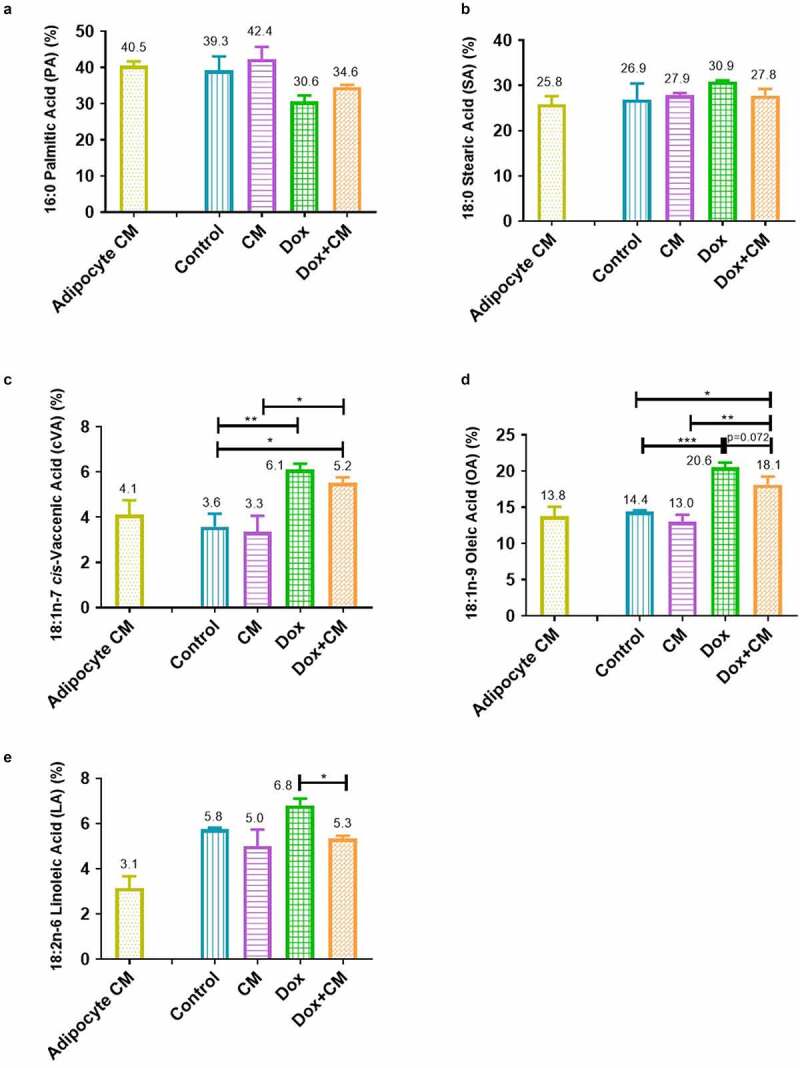
Individual FFA (% of total µg FFA) in treatment-conditioned media of MDA-MB-231 triple negative breast cancer cells treated with/without adipocyte-conditioned media and/or doxorubicin for 48-hours. (a) Palmitic Acid (PA), (b) Stearic Acid (SA), (c) *cis-*Vaccenic Acid (cVA), (d) Oleic Acid (OA), and (e) Linoleic Acid (LA). Results are presented as mean ± SEM (n = 3). One-way ANOVA with Fishers LSD *post hoc* correction was employed. p < 0.05 was considered as statistically significant. * = p < 0.05, ** = p < 0.01 and *** = p < 0.001. Adipocyte CM; 100% Adipocyte-conditioned media; Control, normal growth media; CM, 30% adipocyte-conditioned media and 70% growth media ratio; Dox, 2.5 µM Doxorubicin; Dox+CM, 2.5 µM Doxorubicin + CM (30% adipocyte-conditioned media and 70% growth media ratio). Value above bars represents the mean value

### Statistical Analysis

Graphpad Prism version 7 (Inc, USA) was used for all statistical analysis. Normality was assessed using the Shapiro–Wilks test, and results were reported as means ± SEM. A one- or two-way ANOVA was used to describe differences between three or more groups, followed by the Fisher’s LSD *post hoc* test. Statistical significance was accepted at p < 0.05.

## RESULTS

### Time-Dependent Increase in Cell-Viability after Adipocyte-Conditioned Media Treatment on Doxorubicin-Treated Triple Negative Breast Cancer Cells

Human adipose tissue-derived stem cells (hADSC; Supplementary Figure 2a) were successfully differentiated into mature adipocytes (Supplementary Figure 2b), indicative of lipid accumulation as shown by Oil Red O staining. To ascertain the effect of adipocyte-conditioned media on MDA-MB-231 TNBC cells treated either with or without doxorubicin, WST-1 cell-viability assays were performed at 24-hour and 48-hour post-treatment, respectively. After 24 hours of doxorubicin treatment, cell-viability decreased compared to the control-treated cells (Dox *vs* control, p < 0.0001), whereas-viability was reduced in cells cultured in adipocyte-conditioned media containing Doxorubicin compared to those cultured in CM only (Dox+CM *vs* CM, p < 0.0001) (Figure 1). Following 48 hours of treatment there was a significant increase in cell-viability of Dox+CM treated compared to Dox treated cells (Dox+CM *vs* Dox, p < 0.0001, Figure 1). Consequently, the treatment effect for all experiments was assessed at 48-hour post-treatment.

### Doxorubicin-Induced Apoptosis is not Attenuated by Adipocyte-Conditioned Media Treatment in Triple Negative Breast Cancer Cells

To evaluate whether adipocyte-conditioned media could alter doxorubicin-induced apoptosis, we assessed the protein expression of poly (ADP-ribose) polymerase (PARP) activation (116 kDa) and cleavage (89 kDa), total Caspase-3 (35 kDa) and cleaved caspase 3 (15 kDa).

Following 48 hours of doxorubicin treatment, cleaved-PARP protein expression was increased in CM treated cells (CM *vs* Dox+CM, p < 0.05; Figure 2a). Doxorubicin treatment also increased cleaved-caspase-3 protein expression when compared to the control group (p < 0.05; Figure 2b), confirming that doxorubicin treatment-induced apoptosis in MDA-MB-231 TNBC cells. Cleaved-caspase-3 expression was decreased in the Dox+CM-treated group compared to Dox treatment; however, this was not found to be significant (Figure 2b). The latter suggests that adipocyte-conditioned media did not decrease doxorubicin’s efficacy to induce apoptosis, which did not correlate with the increased cell-viability observed in Dox+CM-treated MDA-MB-231 cells.

### Signalling Pathways not induced by Adipocyte-Conditioned Media in Doxorubicin-Treated Breast Cancer Cells

To ascertain if the activation of cellular proliferation pathways could explain the Doxorubicin treatment resistance as observed with our cell-viability results (increased cell-viability in Dox+CM), protein expression of various cellular proliferation pathways, i.e. PI3K/Akt, ERK and nuclear factor kappa B-p65 (NFĸB-p65) was assessed.

Phosphorylated PI3K (Tyr 467 and Tyr 199) protein expression was increased in CM-treated cells compared to both the control (p < 0.05) and Dox+CM-treated cells (p < 0.05) (Figure 3a). Phosphorylated Akt (pAkt, Ser 473) protein expression was increased in CM-treated (p < 0.001) and decreased in Dox-treated cells (p < 0.05) compared to control cells (Figure 3b). A decrease was observed in Dox+CM-treated cells when compared to both control (p < 0.05) and CM-treated cells (p < 0.001) for pAkt (Figure 3b).

No significant differences were observed for Phosphorylated ERK1/2 (pERK1, Thr 202/Tyr 204; pERK2, Thr185/Tyr187) (Figure 3c) and NFĸB-p65 protein expression in the MDA-MB-231 breast cancer cells either treated with, or without doxorubicin (Figure 3d). In summary, adipocyte-conditioned media treatment did not induce a phosphorylation status of proliferative signalling, which did not correlate with the increased cell-viability observed in Dox+CM-treated MDA-MB-231 cells.

### Mesenchymal-to-Epithelial Transition (MET): Partial Phenotype

Conflicting results are reported on the role of adipocyte-derived factors in cancer invasion and metastasis, which are both associated with acquired treatment resistance [20,50]. This is especially true for the complex processes of epithelial-to-mesenchymal transition (EMT) and mesenchymal-to-epithelial transition (MET) [51,52]. As such, we measured markers of EMT in MDA-MB-231 cells treated with adipocyte-conditioned media and doxorubicin, namely E-cadherin (epithelial) and vimentin (mesenchymal). Vimentin protein expression (Figure 4a) was found to be higher in the control group compared to CM-treated cells (p < 0.01) and higher in Dox-treated compared to Dox+CM-treated cells (p < 0.05). No differences were observed for E-cadherin protein expression between any of the respective treatment groups (Figure 4b).

### Hormone Sensitive Lipase Protein Expression is Induced by Adipocyte-Conditioned Media in Doxorubicin-Treated Breast Cancer Cells

Breast cancer cells disrupt normal lipid metabolism by increasing *de novo* synthesis of FA evident by increased expression of various enzymes regulating this pathway, i.e. Acetyl-CoA carboxylase (ACC), fatty acid synthase (FAS) [31–33] and stearoyl CoA-desaturase-1 (SCD-1), which catalyses the conversion of saturated FA (SFA), e.g. palmitic and stearic acid, to palmitoleic and oleic acid [53–55]. Additionally, lipolysis in adipocytes is also disrupted by increasing the expression of lipolytic proteins (i.e. ATGL and HSL) [25]. Therefore, to ascertain whether secretory factors in the adipocyte-conditioned media induce breast cancer cells to dysregulate lipid metabolism, we assessed the protein expression levels of lipid metabolism markers that regulate *de novo* FA synthesis (FAS and SCD-1) and lipolysis (ATGL and HSL) with or without doxorubicin treatment.

No differences were observed for FAS and SCD-1 between any of the experimental treatment groups (Figure 5a and b). Compared to the control group, HSL protein expression was upregulated after Dox treatment (Control *vs* Dox, p < 0.05; Figure 5d), whereas HSL and ATGL protein expression was higher in the Dox+CM treatment group compared to the control group (ATGL, p < 0.05 and HSL, p < 0.001; Figure 5c and d). Additionally, HSL expression was higher in the Dox+CM-treated compared to Dox-treated (p < 0.05) and CM-treated (p < 0.01) cells, respectively (Figure 5d). Thus, secretory factors in the adipocyte-conditioned media increased HSL protein expression in Doxorubicin-treated MDA-MB-231 cells.

### Adipocyte-derived Factors and Doxorubicin Treatment Alters the Treatment-Conditioned Media Free Fatty Acid Composition of MDA-MB-231 Breast Cancer Cells

To determine if secretory factors in the adipocyte-conditioned media can stimulate the utilization or release of FFA by MDA-MB-231 TNBC cells treated with/without doxorubicin, we assessed the FFA composition of collected treatment-conditioned media in all the experimental treatment groups, to assess any alterations within the media FFA profile.

The total µg FFA per 1 ml adipocyte-conditioned media and the treatment-conditioned media are illustrated in Supplementary Figure 3a. Each FA class is expressed as a percentage of the total microgram FFA per 1 ml. Various FFA were identified in the adipocyte-conditioned media ranging from highest (palmitic acid) to lowest (linoleic acid) content (Supplementary Figure 4a).

First, we observed that the percentage of total saturated FA (Σ SFA) was lower in the treatment-conditioned media of the Dox and Dox+CM experimental groups (after 48 hours) when compared to the adipocyte-conditioned media (unexposed to cells and treatments), suggesting the utilization/internalization of these FFA by the MDA-MB-231 cells (supplementary Figure 3). The opposite was observed for total monounsaturated FA (Σ MUFA) percentage, suggesting the release of MUFA by the MDA-MB-231 cells (supplementary Figure 3). However, when comparing only the experimental treatment groups, no differences were observed for Σ SFA (supplementary Figure 3b) and for total polyunsaturated FA (Σ PUFA) (supplementary Figure 3d).

Furthermore, both Dox (p < 0.05) and Dox+CM (p < 0.01) treatment-conditioned media showed higher Σ MUFA content compared to treatment-conditioned media from the control group, whereas Dox+CM showed higher Σ MUFA content compared to the treatment-conditioned media from the CM group (p < 0.01; supplementary Figure 3c). Similar results were observed for both *cis-*vaccenic acid (cVA, 18:1 n-7) and oleic acid (OA, 18:1 n-9) in both Dox (cVA, p < 0.01 and OA, p < 0.001) and Dox+CM (cVA, p < 0.05 and OA, p < 0.05) treatment groups, indicating higher cVA and OA content compared to the control group (Figure 6c and d). The Dox+CM showed higher cVA and OA content compared to the CM group (cVA, p < 0.05 and OA, p < 0.01; Figure 6c and d). A trend (p = 0.072) towards lower OA content in Dox+CM compared to the Dox treatment-conditioned media was observed (Figure 6d). Linoleic acid (LA, 18:2 n-6) was lower in the treatment-conditioned media of the Dox+CM compared to the Dox group (p < 0.05, Figure 6e). Thus, we suggest that doxorubicin treatment increased the release of *cis*-vaccenic acid and oleic acid by MDA-MB-231 cells, whereas secretory factors in adipocyte-conditioned media inhibited the release of linoleic acid by MDA-MB-231 cells treated with doxorubicin.

### Adipocyte-derived Factors Induced the Utilization of Inflammatory Markers by Doxorubicin Treated Breast Cancer Cells Evident within Treatment-conditioned Media

To determine if secretory factors in the adipocyte-conditioned media can alter the release or utilization of inflammatory proteins by MDA-MB-231 TNBC cells treated with doxorubicin, we determined the concentrations of various inflammatory markers in the adipocyte conditioned media (unexposed to cells and treatments) and the collected treatment-conditioned media of all experimental groups (after 48 hours).

**Figure 7.**
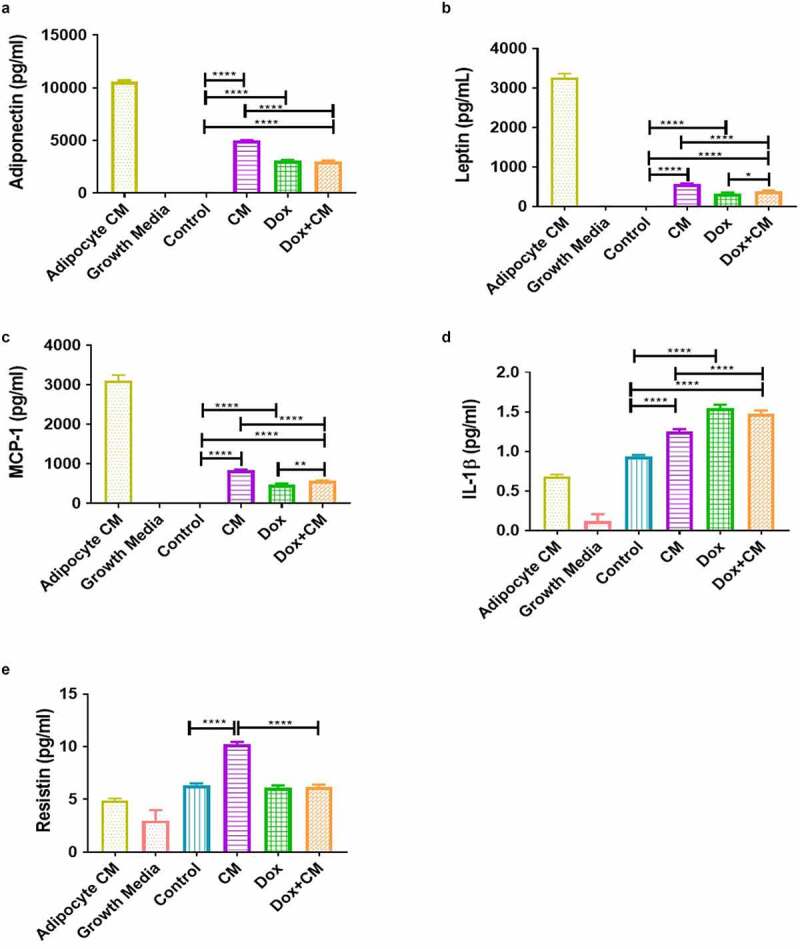
Inflammatory marker concentrations in treatment-conditioned media of MDA-MB-231 triple negative breast cancer cells treated with/without adipocyte-conditioned media and/or Dox for 48-hours. (a) Adiponectin, (b) Leptin, (c) MCP-1, (d) IL-1β, and (e) Resistin. Results are presented as mean ± SEM (n = 4). One-way ANOVA with Fishers LSD *post hoc* correction was employed. p < 0.05 was considered as statistically significant. * = p < 0.05, ** = p < 0.01 and **** = p < 0.0001. Adipocyte CM, 100% Adipocyte-conditioned media; Control, normal growth media; CM, 30% adipocyte-conditioned media and 70% growth media ratio; Dox, 2.5 µM Doxorubicin; Dox+CM, 2.5 µM Doxorubicin + CM (30% adipocyte-conditioned media and 70% growth media ratio)

Various adipokines (adiponectin, leptin, MCP-1, resistin, and IL-1β) were identified in the adipocyte-conditioned media ranging from highest (adiponectin) to lowest (IL-1β) concentration (Supplementary Figure 4b). First, we observed that adiponectin, leptin and MCP-1 concentrations were lower in the treatment-conditioned media of all experimental groups (after 48 hours) when compared to the adipocyte-conditioned media (unexposed to cells and treatments), indicating the utilization/internalization of these adipokines by the MDA-MB-231 TNBC cells. The opposite was observed for IL-1β and resistin concentrations, indicating the release of these adipokines by the MDA-MB-231 cells (Figure 7).

When comparing only the experimental treatment groups, adiponectin, leptin, MCP-1, and IL-1β concentrations in the treatment-conditioned media were found to be higher in the CM, Dox and Dox+CM groups compared to the control group (all p < 0.0001; Figure 7). Similarly, adiponectin, leptin and MCP-1 concentrations were higher in the CM compared to the Dox+CM group (p < 0.0001; Figure 7), whereas the opposite was observed for IL-1β (p < 0.0001; Figure 7d). Both leptin (p < 0.05) and MCP-1 (p < 0.01) concentrations were higher in the Dox+CM compared to the Dox-treated group (Figure 7b and c). Resistin concentration was higher in the CM group compared to both the control and Dox+CM groups (p < 0.0001; Figure 7e). These results suggest that secretory factors in the adipocyte-conditioned media increased the utilization/internalization of leptin and MCP-1 proteins by MDA-MB-231 cells treated with doxorubicin.

## DISCUSSION

### Doxorubicin-induced Apoptosis is Not Attenuated in Triple Negative Breast Cancer Cells Treated with Adipocyte Secretory Factors

Doxorubicin induces DNA damage, generates ROS, initiates mitochondrial membrane dysfunction, and induces apoptosis in response to topoisomerase II inhibition to promote apoptotic cell death in cancer cells through activation of caspases [6,56,57]. Doxorubicin decreases cell-viability, upregulates caspase-3 and PARP activation (repair damaged DNA) in a time-dependent manner (48–72 hours post treatment) in MDA-MB-231 cells [58–61]. Additionally, adipocytes and/or adipocyte-derived factors can promote Daunorubicin treatment resistance through upregulation of anti-apoptotic bcl-2, and downregulation of pro-apoptotic bad and pim-2 family members [62,63]. Doxorubicin-induced apoptosis was suppressed in MDA-MB-231 cells by resistin induced autophagy [28]. Here, we did not observe Doxorubicin-induced Caspase-3 and PARP activation by adipocyte-conditioned media, which did not correlate with the increased cell-viability observed in Dox+CM-treated cells. Although previous studies have linked alterations in cell death pathways as the main underlying mechanism responsible for adipocyte-induced-drug resistance [28,62,64], it is plausible that adipocyte secretory factors can induce treatment resistance *via* other mechanisms of action.

Other cancer drug resistance mechanisms linked to adipocyte and/or adipocyte-derived factors include, but are not limited to, alterations in drug uptake (decreased), efflux (increased) and alterations in drug transporter proteins [29,63], increased drug sequestering [65], alterations in DNA repair mechanisms/signalling/cell cycle pathways [66], as well as metabolic lipid reprogramming [10,25,36], highlighting the complexity of drug resistance.

Increased leptin, IL-8, IL-6, and IL-1β concentrations is reported in MDA-MB-231 and MDA-MB-468 cells after co-cultivation with adipocytes or adipocyte-conditioned medium favouring tumour cell survival and progression [20,67]. These adipocyte secretory factors (IL-6, TNF-α and leptin) were identified in acquired breast cancer drug resistance [9,68]. Leptin derived from obese adipocytes attenuated Tamoxifen’s® treatment efficacy [68], whereas MCP-1 protected breast cancer cells from 5-Fluorouracil [69]. Trastuzumab® antibody-mediated dependent cellular cytotoxicity was inhibited by adipocyte-derived factors in breast cancer cells [70], which was corroborated by 66, for Lapatinib (tyrosine kinase inhibitor). Therefore, we propose that the treatment resistance observed in the Dox+CM group (increased cell-viability) may in part be as a result of secretory factors in the adipocyte-conditioned media stimulating the utilization/internalization of leptin and MCP-1 proteins by doxorubicin-treated MDA-MB-231 TNBC cells. The latter may lead to integrated actions, which promote macrophage recruitment, increasing synthesis of pro-inflammatory mediators (e.g. leptin) and anti-inflammatory cytokines, exacerbating local inflammation and inducing tissue repair in the tumour microenvironment, respectively [19,71,72]. The recruitment of immune cells to the tumour microenvironment stimulates the secretion of matrix metalloproteinase-9 (role in matrix degradation) to evade the host’s immune responses and induces angiogenesis [73], increasing cell-viability, metastasis, and ultimately creating favourable conditions to develop treatment resistance [74].

### Adipocyte Secretory Factors Favours a Partial Mesenchymal-to-Epithelial Transition but Does Not Activate Signalling Pathways in Doxorubicin-Treated TNBC Cells

Adipocytes also induce signalling pathways in breast cancer cells that facilitate proliferation, invasion and metastasis, which is associated with acquired treatment resistance [8,20,50,75]. Metastasis is a complex process, which includes cells migrating through the extracellular matrix as a result of epithelial-to-mesenchymal transition (EMT) [51] to facilitate the migration of cancer cells away from the primary tumour, as well as mesenchymal-to-epithelial transition (MET) to form tumours at distant sites [52].

Adipocytes induce morphological changes (loss of stellate and gain of round/mass colony phenotype), and changes the MET protein profile (increased E-cadherin and Claudin-7 expression) in mesenchymal MDA-MB-231 cells [23, 75, 76]. Adipocyte-conditioned media only affects cell morphology, but not MET biomarkers, suggestive of a partial MET protein profile in MDA-MB-231 cells [76]. We reason that a partial MET profile may be plausible for our findings since decreased expression of the cytoskeletal structural protein, vimentin, was induced by adipocyte-conditioned media with or without doxorubicin treatment. Normally, high vimentin protein expression is observed in mesenchymal MDA-MB-231 cells [76], supporting our findings. The cell surface protein, E-cadherin, did not change and is in agreement with MDA-MB-231 cells co-cultured with adipocytes showing low protein expression of E-cadherin [23].

Reports on how adipocyte-derived factors induce EMT and MET remain conflicting [50,77,78]. Of relevance, 71, reported higher MCP-1 levels, which are associated with cell invasion and metastasis in TNBC cells (BT549 and HCC1395) by inducing the p44/42 MAP kinase pathway [71]. Conditioned medium from human mammary cancer-associated adipocytes increased MCP-1 levels, which decreased vimentin expression in MDA-MB-231 cells [19]; this is in agreement with the increased concentration of MCP-1 in treatment-conditioned media and decreased vimentin protein expression observed in the Dox+CM-treated compared to the Dox-treated group in our current *in vitro* model.

Similar to the findings reported by others [66,79], we did not observe any differences in the proliferation signalling pathways (i.e. PI3K, Akt, ERK1/2 and its phosphorylation, and NFĸB-p65) between the Dox and Dox+CM-treated MDA-MB-231 cells. It is plausible that adipocyte secretory factors (resistin, TGF-β, IL-6, leptin and IL-8) can induce MET *via* an additional signalling pathway, such as STAT-3 [22,50,78]. Adipocyte secretory factors can also induce partial MET *via* other mechanisms of action, such as lipid accumulation resulting from increased uptake/utilization of adipocyte-derived FFA, which lead to the uncoupling of FA oxidation favouring invasion due to EMT, but not the activation of proliferation pathways [42].

### Adipocyte-Derived Factors Increased Hormone Sensitive Lipase Protein Expression and Altered the FFA Composition by Doxorubicin-Treated MDA-MB-231 Cells

Lipid metabolic reprogramming is evident in breast cancer cells showing a strong dependence on both *de novo* synthesis of FA and the uptake of adipocyte-derived exogenous FFA to sustain its high-energy demand *via* β-oxidation [25,36,42]. Additionally, these excess FFA confer to a more densely packed membrane with structural lipids (phospholipids/cholesteryl esters) [30] and are also esterified into triglycerides and stored in lipid droplets within breast cancer cells [80], all of which have been shown to protect cancer cells from the cytotoxic effects of chemotherapeutic agents [10]. Here, HSL protein expression was upregulated by adipocyte-conditioned media in doxorubicin-treated MDA-MB-231 cells. This is in agreement with previous work showing breast cancer cells inducing adipocytes to release FFA through altered ATGL and HSL expression [10,25,34].

Evidence also showed that chemotherapeutic agents 5-Fluorouracil and Irinotecan decreased the expression of lipogenesis (ACC and FAS) and lipolysis (HSL) and also decreased the SFAs (PA) and MUFAs (PTA) content, thereby inducing cancer cell invasion and metastasis by increasing lipid accumulation [81]. Doxorubicin and 5-Fluorouracil treatment increased the number of lipid droplets, increased the SFAs (PA) and PUFAs content, and decreased the MUFAs (OA and PTA) in the membrane phospholipids of HepG2 cancer cells [82]. Of relevance, MDA-MB-231 cells also accumulate more triglyceride-rich lipid droplets due to increased partitioning of FFA into triglycerides compared to MCF-7 cells [25]. Therefore, in agreement with our results, it is plausible that lipid enriched TNBC cells may be as a result of altered HSL protein expression, mitochondrial dysfunction or the inhibition of mitochondrial FA oxidation induced by Doxorubicin (increased ROS), which shifts the utilization of FA away from oxidation and indirectly promotes triglyceride synthesis and subsequent lipid storage (triglyceride-rich lipid droplets) or more densely packed membranes in tumour cells [81–83].

We further propose that adipocyte-derived factors may induce MDA-MB-231 TNBC cells to redirect and incorporate n-6 PUFAs into lipid droplets (PUFA-TAGs) rather than membrane structures in order to limit doxorubicin-induced toxicity [84], by inhibiting the release of LA, as suggested by the decreased LA observed content within Dox+CM FFA media fraction. However, the opposite scenario may also be possible where adipocyte secretory factors induce the utilization/internalization of linoleic acid by MDA-MB-231 cells treated with doxorubicin (Dox+CM) causing the lower media content. This is based on the fact that we did not determine the fresh growth media FFA profiles; therefore, we can only interpret at best our results as a suggestion of what may happen in both suggested scenarios.

Linoleic acid (LA; 18:2 n-6) is an essential FA [85], which is subjected to desaturation (FA desaturases), as well as elongation (Elovl2 and Elovl5) to form other major long-chain n-6 PUFA, such as arachidonic acid (AA; 20:4 n-6), which is well known for its pro-inflammatory and pro-carcinogenic effects in breast cancer [85]. The pro-inflammatory effects of n-6 PUFA are due to the diversity of functions associated with lipid-derived bioactive mediators, such as eicosanoids, prostaglandins, leukotrienes and thromboxanes [86]. These lipid-derived bioactive mediators also act as second messengers in cellular signalling [87], regulating angiogenesis, proliferation, migration, metastasis and inflammation [88], promoting an ideal microenvironment that facilitates carcinogenesis and acquired cancer treatment resistance.

To summarize, we provide evidence that adipocyte-derived factors induced doxorubicin treatment resistance in an *in vitro* adipocyte-conditioned media model. The increased cell viability may in part be due to adipocyte secretory factors inducing increased HSL protein expression in doxorubicin-treated MDA-MB-231 TNBC cells, including the increased utilization of inflammatory mediators (leptin and MCP-1) and possibly the inhibition of FFA release (decreased n-6 PUFA, LA).

Lastly, the present study was limited by the following. The adipocyte-conditioned media did not induce apoptosis or a proliferative state and could therefore not account for the increased cell-viability observed in doxorubicin-treated MDA-MB-231 cells. Considering this, it should be highlighted that the apoptosis (PARP and Caspase-3) and proliferation signalling pathways assessed (PI3K, Akt, ERK1/2 and NFĸB-p65), could have been activated at earlier time points (i.e. 12, 18 or 24 hours) which we did not assess; this could probably also explain the increased cell-viability observed at 48 hours. We therefore propose that apoptosis and proliferation signalling pathways analysis should be assessed at these earlier time points. In addition, the FFA profile of both the normal growth media (DMEM, 10% FBS and 1% PenStrep) and adipocyte-conditioned media (30% adipocyte-conditioned media and 70% normal growth media ratio) unexposed to cells/treatments was not determined, which for future investigations should be done to serve as additional internal controls. For future investigations, we also propose to assess the phospholipid-FA and FFA profiles of the MDA-MB-231 cells themselves, which will be more informative in terms of intracellular FA uptake, utilization and release.

## CONCLUSION

**Figure 8.**
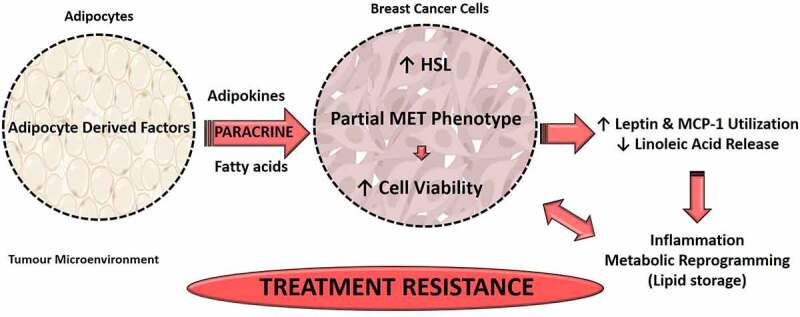
Summary of the *in vitro* model findings. HSL, Hormone sensitive lipase; MCP-1, macrophage chemoattractant protein-1; MET, mesenchymal-to-epithelial transition

We highlight the complex role that adipocyte-derived factors play in doxorubicin treatment resistance in TNBC cells within tumour microenvironment. Adipocyte secretory factors induced doxorubicin treatment resistance (increased cell-viability), but not *via* induction apoptosis or cellular proliferation. Here we identified other adipocyte-derived mechanisms underlying treatment resistance, whereby adipocyte secretory factors induced a partial MET phenotype and induced hormone-sensitive lipase protein expression in doxorubicin-treated MDA-MB-231 cells in a paracrine manner. Adipocyte-conditioned media also increased the utilization of inflammatory mediators (leptin and MCP-1) and possibly inhibited the release of FFA (decreased linoleic acid) by doxorubicin-treated MDA-MB-231 cells. This confers to acquired treatment resistance by promoting survival mechanisms including inflammation and metabolic reprogramming (lipid storage) in order to evade the toxic effects induced by doxorubicin (Figure 8).

## Supplementary Material

Supplemental Material

## Data Availability

The data that support the findings of this study are openly available in figshare at http://doi.org/[10.6084/m9.figshare.16577759] or https://figshare.com/s/97ebbb7b329b8a642853.

## Notes

**Abbreviations**: Σ MUFA, Total monounsaturated fatty acids; Σ PUFA, Total polyunsaturated fatty acids; Σ SFA, Total saturated fatty acids; ACC, Acetyl-CoA carboxylase; ATGL, Adipose triglyceride lipase; AM, Adipogenic differentiation medium; CD36, Fatty acid translocase; CM, conditioned media treatment group; CMS, Chloroform:Methanol:Saline; cVA, cis-Vaccenic acid (18:1n-7); DMEM, Dulbecco’s Modified Eagle’s medium; Dox, Doxorubicin treatment group; Dox+CM, Combination conditioned media and doxorubicin treatment group; EMT, Epithelial-to-mesenchymal transition; FA, Fatty acids; FABP-4, Fatty acid-binding protein-4; FABP-5, Fatty acid-binding protein-5; FAMEs, Fatty acid methyl esters; FAS, Fatty acid synthase; FBS, Foetal bovine serum; FFA, Free fatty acids (FFA); GLC, Gas-liquid chromatography; hADSC, Human adipose tissue-derived stem cell; HSL, Hormone sensitive lipase; IBMX, 3-Isobutyl-1-methylxanthine; IL-1β, interleukin-1β; IL-6, interleukin-6; LA, Linoleic acid (18:2n-6); MAPK, MAP kinase pathway; MCP-1, Macrophage chemoattractant protein-1; MET, mesenchymal-to-epithelial transition; NFκB, Nuclear factor kappa B; OA, Oleic acid (18:1n-9); PA, Palmitic Acid (C16:0); PARP, poly (ADP-ribose) polymerase; PBS, Phosphate buffered saline; PenStrep, Penicillin Streptomycin; PI3K, Phosphoinositide-3-kinase; RIPA, Radio-immunoprecipitation assay buffer; SA, Stearic Acid (C18:0); SCD-1, Stearoyl CoA-desaturase-1; SEM, Standard error of the mean; SFA, Saturated fatty acids; STAT-3, Signal transducer and activator of transcription-3; TAG, Triacylglycerol; TBS-T, Tris Buffered Saline-Tween 20; TLC, thin-layer chromatography; TNBC, Triple negative breast cancer; TNF-α, Tumour necrosis factor-alpha

## HIGHLIGHTS

- Effect of secretory factors in adipocyte-conditioned media on doxorubicin-treated MDA-MB-231 cells was investigated.
- Hormone sensitive lipase was increased in doxorubicin-treated cells.
- Adipocyte-conditioned media increased utilization of inflammatory mediators by doxorubicin-treated cells.
- Adipocyte-conditioned media inhibited the release of free fatty acids by doxorubicin-treated cells.

## Submission declaration:

We confirm that this work is original and has not been published elsewhere, nor is it currently under consideration for publication elsewhere.
