# Supplementary material for: The paracrine effects of adipocytes on lipid metabolism in doxorubicin-treated triple negative breast cancer cells
Source: Adipocyte. 2021 Nov 23;10(1):505–23. doi: 10.1080/21623945.2021.1979758 (PMC8632082; doi:10.1080/21623945.2021.1979758)
Supplement: Supplemental Material [file KADI_A_1979758_SM2755.docx]

**Supplementary Table** **1:** Antibodies used in *in vitro* model for western blotting.

| Primary Antibody | Size | Concentration | Company |
| --- | --- | --- | --- |
| Apoptosis | | |  |
| PARP and Cleaved-PARP | 89, 116 kDa | 1:1000 | Cell Signalling (#9532) |
| Caspase-3 and Cleaved-caspase 3 | 35, 17 kDa | 1:1000 | Cell Signalling (# 9662) |
| Proliferation | | |  |
| Total PI3K | 85 kDa | 1:1000 | Abcam (#ab86714) |
| Phosphorylated PI3K p85/p55 (Tyr 467/Tyr 199) | 85,55 kDa | 1:1000 | Elabscience (#ENP0224) |
| Total AKT | 60 kDa | 1:1000 | Cell Signalling (#9272) |
| Phosphorylated AKT (Ser 473) | 60 kDa | 1:1000 | Cell Signalling (#4060) |
| Total ERK 1/2 | 44,42 kDa | 1:1000 | Abcam (#ab184699) |
| Phosphorylated ERK1/2 (Thr 202/Tyr 214, Thr 185/Tyr 187) | 44,42 kDa | 1:2000 | Cell Signalling (#4370) |
| Epithelial -to-Mesenchymal Transition (EMT) | | |  |
| Snail | 29 kDa | 1:1000 | Cell Signalling (#3879) |
| Vimentin | 57 kDa | 1:1000 | Cell Signalling (#5741) |
| E-cadherin | 135 kDa | 1:1000 | Cell Signalling (#3195) |
| Lipid Metabolism | | |  |
| Fatty acid synthase (FAS) | 273 kDa | 1:1000 | Cell Signalling (#3180) |
| Stearoyl-CoA desaturase-1 (SCD-1) | 37 kDa | 1:1000 | Cell Signalling (#2794) |
| Adipose tissue triglyceride lipase (ATGL) | 54 kDa | 1:1000 | Cell Signalling (# 2439) |
| Nuclear factor kappa B (NFĸB-p65) | 65 kDa | 1:1000 | Cell Signalling (#8242) |
| Hormone sensitive lipase (HSL) | 83 kDa | 1:1000 | Cell Signalling (# 4107) |
| Secondary Antibodies | | | |
| Anti-mouse |  | 1:10 000 | Cell Signalling (#7076S) |
| Anti-rabbit |  | 1:10 000 | Cell Signalling (#7074) |

**
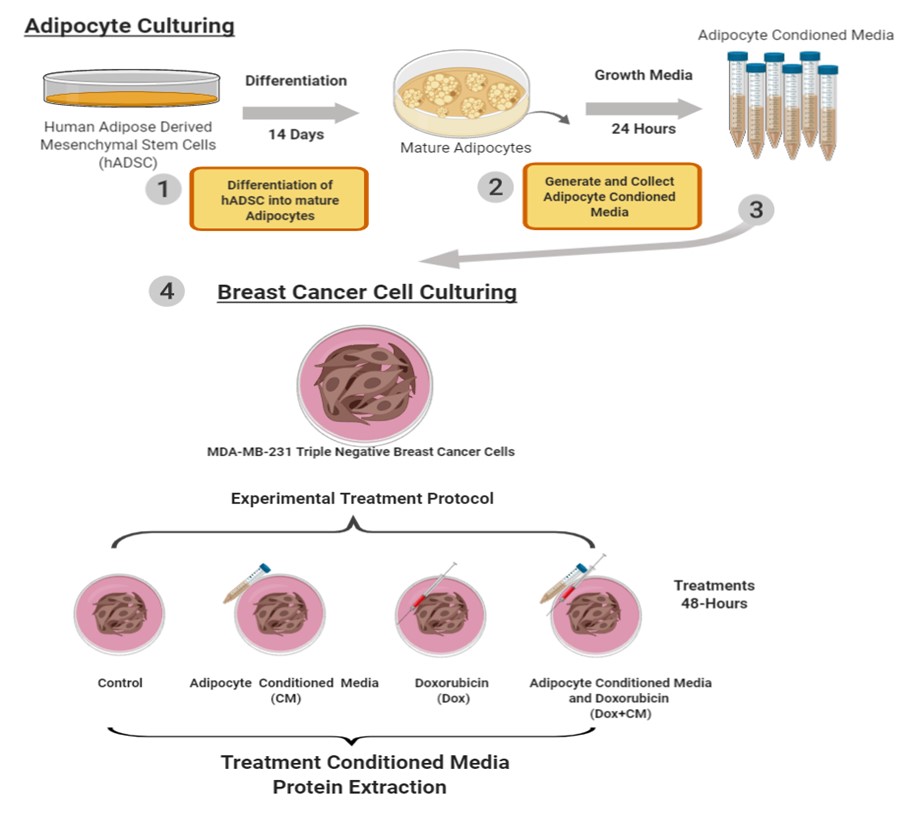
**

**Supplementary Figure 1**: Outline of the *in vitro* model experimental treatment protocol (created with BioRender.com^®^). Control, normal growth media; CM, 30% adipocyte-conditioned media and 70% growth media ratio; Dox, 2.5µM Doxorubicin; Dox+CM, 2.5µM Doxorubicin + CM (30% adipocyte-conditioned media and 70% growth media ratio).


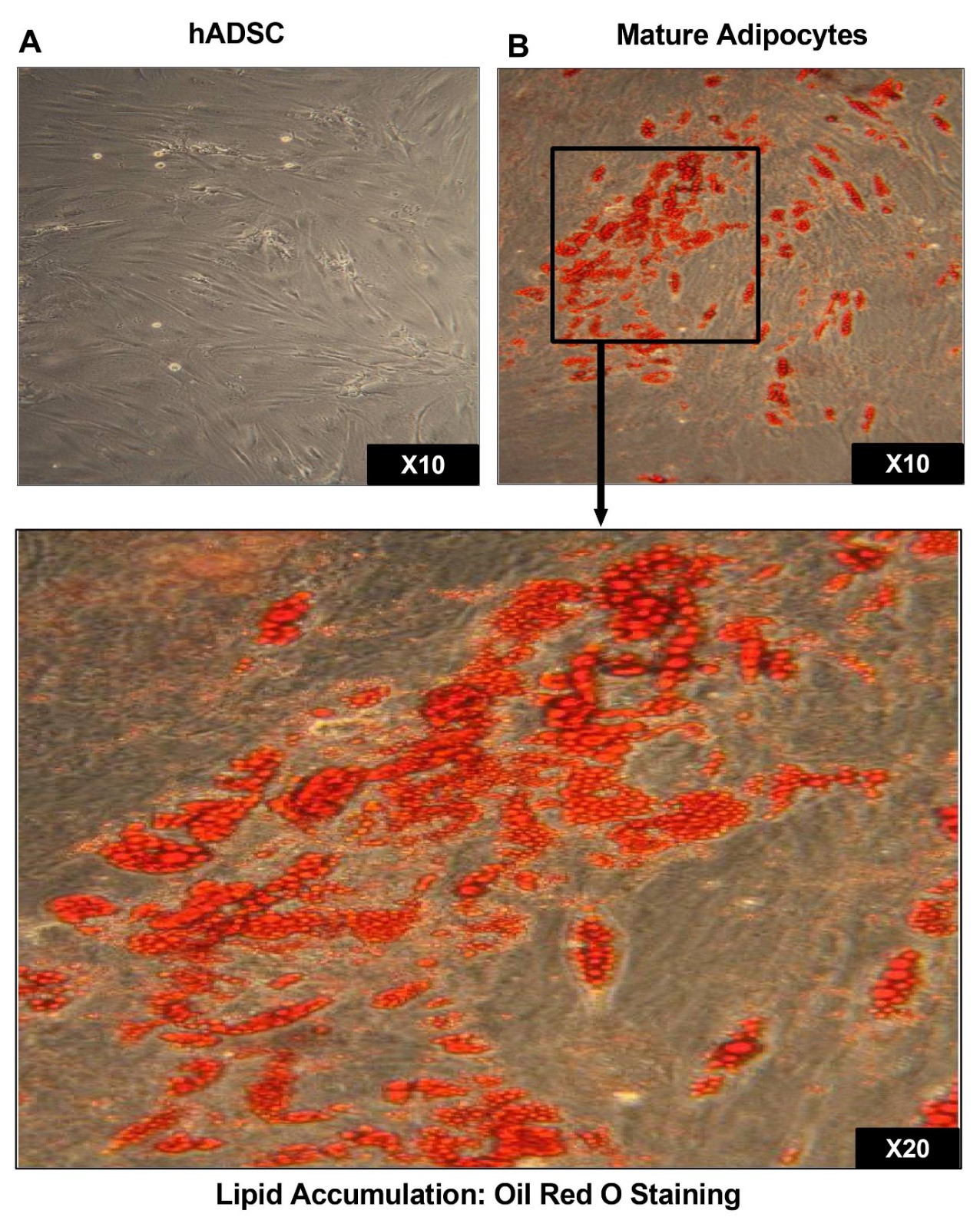


**Supplementary Figure 2**: (A) Human adipose tissue derived stem cell differentiation into mature adipocytes. (B) Lipid accumulation in mature adipocytes (n=6) assessed with Oil Red O staining (day 14). hADSC, human adipose tissue derived stem cells.

**Supplementary Figure 3**: The FFA class percentage composition in 100% adipocyte-conditioned media (Adipocyte-CM) and collected treatment-conditioned media of all experimental treatment groups. (A) Total FFA µg/mL, (B) Total SFAs (%), (C) Total MUFAs (%) and (D) Total PUFAs (%). Results are presented as mean ± SEM (n=3). One-way ANOVA with Fishers LSD *post hoc* correction was employed. p<0.05 was considered as statistically significant. * = p<0.05, ** = p<0.01 and *** = p<0.001. Control, normal growth media; CM, 30% adipocyte-conditioned media and 70% growth media ratio; Dox, 2.5µM Doxorubicin; Dox+CM, 2.5µM Doxorubicin + CM (30% adipocyte-conditioned media and 70% growth media ratio). FFA, free fatty acids; MUFA, monounsaturated fatty acids; PUFA, polyunsaturated fatty acids; SFA, saturated fatty acids. Value above bars represents the mean value.

**Supplementary Figure 4:** (A) Free fatty acid composition (%) and (B) Adipokine concentrations in 100 % adipocyte-conditioned media. Results are presented as mean ± SEM (n=4). Value above bars represents the mean value.
